# Editorial: Perspectives on crop response to abiotic stresses: function of lipid components

**DOI:** 10.3389/fpls.2025.1648015

**Published:** 2025-07-08

**Authors:** Feng Liu

**Affiliations:** Key Laboratory of Oasis Eco-agriculture, College of Agriculture, Shihezi University, Shihezi, Xinjiang, China

**Keywords:** lipid metabolism, abiotic stress, membrane lipids, fatty acid unsaturation, omics approaches, crop tolerance

Abiotic stresses-drought, salinity, heat, and cold- pose persistent and escalating threats to global agricultural productivity and food security. While their detrimental impact on crop yield and quality is universal, the inherent sensitivity and resilience mechanisms vary dramatically not only between species but, critically, between varieties of the same crop. Unraveling the genetic and physiological basis of these varietal differences is paramount for developing the next generation of stress-tolerant crops through targeted breeding. Amid the complex orchestra of plant stress responses, lipid metabolism emerges as a pivotal, yet underexplored conductor. Lipids transcend their role as mere structural membrane components; they function as dynamic signaling molecules, energy reservoirs, modulators of protein activity, and rapid responders to environmental cues. Their composition and abundance provide a real-time, molecular fingerprint of a plant’s physiological state under stress.

Despite their fundamental importance, research into the intricate landscape of crop lipids has progressed slowly. The sheer diversity and structural complexity of lipid species, coupled with the challenges of analyzing them within complex plant matrices, have hindered the construction of comprehensive metabolic networks and a deep understanding of their functional regulation in an agricultural context. Crucially, the question of *how* sp*ecific lipid components contribute to the differential stress sensitivity among crop varieties* remains largely unanswered. Does the lipid “signature” of a tolerant cultivar differ fundamentally from a sensitive one? Which lipid classes or individual species act as key sentinels or mediators of resilience? Deciphering this “lipid language” of varietal tolerance lies at the core challenge this Research Topic aimed to address.

We envisioned this Research Topic as a platform to propel research beyond cataloging stress-induced lipid changes. Our goal was to foster studies that: Develop and Refine Tools: Advance methodologies for accurate, high-throughput identification and quantification of the diverse lipidome in agriculturally relevant crops. Map Dynamic Responses: Characterize the spatio-temporal alterations in lipid components and abundance triggered by specific abiotic stresses. Elucidate Physiological Roles: Define the causal physiological functions of specific lipid classes or molecules in conferring tolerance mechanisms (e.g., membrane stability, signaling cascades, antioxidant defense, energy homeostasis). Uncover Molecular Control: Decipher the genetic and regulatory networks governing lipid metabolic flux and remodeling under stress, including varietal differences in gene expression and enzyme activity.

This Research Topic brings together four contributions that collectively advance our understanding of lipids in crop abiotic stress responses. Wei et al. investigate phospholipase C (PLC) inhibition in maize, revealing that disrupted PLC activity alters phospholipid and glycolipid profiles, particularly increasing phosphatidylcholine (PC) and phosphatidylethanolamine (PE) while decreasing monogalactosyldiacylglycerol (MGDG). This remodeling enhances membrane stability under stress, with transcriptomic data showing upregulation of *de novo* lipid biosynthesis pathways. The study underscores PLC’s role in balancing lipid catabolism and anabolism, a process critical for maintaining chloroplast integrity and photosynthetic efficiency. Dhaliwal et al. demonstrate that cotton seeds with higher unsaturated fatty acid content exhibit superior cold germination. Lines with elevated linoleic acid (C18:2) and reduced palmitic acid (C16:0) maintain membrane fluidity at low temperatures, as evidenced by lipidome profiling showing increased unsaturation in phospholipids like phosphatidylglycerol (PG). This work links fatty acid composition directly to stress tolerance, emphasizing the importance of membrane physical properties in germination under cold stress. Zhao et al. identify Glyceraldehyde-3-phosphate dehydrogenase (GAPDH) family genes in soybean, showing that GmGAPDH14 enhances salt tolerance by reducing lipid peroxidation and increasing superoxide dismutase activity. The study highlights how GAPDH-mediated glycolytic pathways intersect with lipid metabolism, providing energy and redox balance to counteract oxidative damage. This molecular link between carbohydrate and lipid metabolism underscores their collaborative role in stress adaptation. Tietel et al. explore nitrogen (N) fertilization effects on jojoba wax, revealing that N availability modulates fatty alcohol composition and antioxidant content (e.g., tocopherols, phytosterols). Elevated N alters wax ester profiles, with quadratic trends in fatty alcohol unsaturation influencing membrane stability and stress resistance. This work bridges agricultural management practices with lipid metabolism, showing how nutrient inputs can be optimized to enhance crop stress tolerance.

While these studies advance our understanding, several frontiers remain: How do specific lipid species (e.g., lysophospholipids, oxylipins) act as signaling molecules in stress pathways? Can lipid metabolic networks be engineered for tolerance to multiple stresses (e.g., drought and heat)? How can lipid-based markers (e.g., unsaturation ratios, antioxidant levels) be used for high-throughput breeding of stress-tolerant varieties? How do climate variables (e.g., CO_2_, temperature) intersect with lipid metabolism to shape stress responses?

This Research Topic reinforces lipid metabolism as a central node in crop abiotic stress responses, with implications for both basic science and agricultural innovation. By integrating molecular, physiological, and agronomic perspectives, these studies pave the way for targeted breeding strategies and management practices that leverage lipid-mediated tolerance. Future research should prioritize functional validation of lipid regulators, multi-omics modeling of metabolic networks, and field-scale validation of lipid-based stress markers, ensuring sustainable solutions for climate-resilient agriculture. Understanding the “lipid signatures” of tolerance specific to elite cultivars provides invaluable biomarkers and targets for breeders. As we move forward, integrating deep lipid phenotyping with genomics, transcriptomics, and precise physiological characterization across diverse germplasm will be essential. The goal remains clear: to translate the intricate language of lipids into actionable knowledge for designing crops equipped with enhanced, lipid-mediated resilience to conquer the challenges of a changing climate.

